# Sagittal alignment of the cervical spine: radiographic analysis of 111 asymptomatic adolescents, a retrospective observational study

**DOI:** 10.1186/s12891-022-05792-x

**Published:** 2022-09-03

**Authors:** Yanjie Zhu, Xinkun Zhang, Yunshan Fan, Zhi Zhou, Guangfei Gu, Chuanfeng Wang, Chaobo Feng, Jia Chen, Shisheng He, Haijian Ni

**Affiliations:** 1 Orthopedic Department, Shanghai Tenth People’s Hospital, Tongji University School of Medicine, Shanghai, China; 2 Spinal Pain Research Institute, Tongji University School of Medicine, Shanghai, China

**Keywords:** Cervical morphology, Sagittal alignment parameter, Asymptomatic adolescent, C2-7 Cobb, C7 Slope, C2-7 plumb line

## Abstract

**Purpose:**

To describe the cervical spine morphology and explore its relationship to global sagittal alignment parameters in the asymptomatic adolescent population.

**Methods:**

A total of 111 adolescent subjects were included. Sagittal alignment parameters, including C7 Slope, C2-C7 Cobb, C2-7 plumb line (PL), C2-S1 Sagittal Vertical Axis (SVA), C7-S1 SVA, T5-12 Cobb, T10-L2 Cobb, L1-S1 Cobb, pelvic incidence (PI), pelvic tilt (PT) and sacral slope (SS), were obtained from lateral radiographs.

**Results:**

Forty-four males and sixty-seven females with a mean age of 16.12 ± 2.40 years were included in this study. The mean values of C7 Slope, C2-7 Cobb and C2-7PL were 20.45 ± 8.88°, -7.72 ± 12.10°, and 13.53 ± 11.63 mm, respectively. C2-7 Cobb, C7 Slope showed significant differences between the male and female groups. Correlation analysis showed that C7 slope was significantly correlated with C2-7 Cobb (*r* = -0.544, *P* < 0.001), C2-S1 SVA (*r* = 0.335, *P* < 0.001), and C7-S1 SVA (*r* = 0.310, *P* = 0.001), but not lumbosacral parameters(L5-S1 Cobb, PI, PT, SS). Using a modified method of Toyama to describe the cervical spine morphology, there were 37 cases (33.3%) in the Lordotic group, and C7 slope, C2-7 Cobb and C2-7PL showed significant differences between groups. According to C2-C7 Cobb, there were 80 Lordotic cases (72.1%). C7 slope and C2-7PL were significantly different between the two groups.

**Conclusion:**

The cervical spine morphology of asymptomatic adolescents varies widely, from lordotic to kyphotic. Combining different classification methods provides a better understanding of the morphology of the cervical spine. C7 slope is an important predictor of global sagittal balance and C2-7PL is a key parameter for restoring cervical lordosis, which should be considered pre-operatively and for conservative treatment. Cervical regional sagittal alignment parameters are not correlated with lumbosacral parameters, and C2-7 Cobb, C7 Slope showed significant differences between males and females.

## Introduction

Over the past few decades, an increasing number of studies have focused on cervical sagittal alignment [1–8]. It has been recognized that the normal function of the cervical spine largely relies on the cervical sagittal alignment, while abnormal morphology can cause pain, degeneration, disability and poor operative outcomes [2, 5, 9–13]. However, the definition of “normal” cervical spine morphology remains ambiguous.

Cervical lordosis is considered to be the natural curve as the result of the development and balancing of the thoracic kyphosis [14]. Theoretically, a kyphotic cervical spine puts the musculature of the neck in a more tense state, and the pressure on the intervertebral discs continues to increase, accelerating the deterioration of degeneration and deformity. In postoperative patients, there may also be an impact on fusion rates, and degeneration of adjacent vertebrae [9].

Contrary to these theories, several studies have shown that a non-lordotic cervical spine is common in asymptomatic adults [2, 3, 6, 7, 14]. Nevertheless, these studies could not exclude the effect of degeneration on the natural cervical morphology. Ideally, the spinal sagittal alignment of skeletally mature adolescents can help us better understand the natural morphology of the cervical spine, yet such studies are currently scarce [15]. Hence, the purpose of this study was to describe the cervical sagittal alignment parameters and their relationship to the global spine alignment parameters in asymptomatic adolescent subjects.

## Materials and methods

### Subjects

A total of 111 adolescent subjects (44 males and 67 females), aged 15–20 years (mean age 16.12 ± 2.40 years), who visited our clinic for spinal deformity screening in 2014–2019, were included in this study. All the subjects were in Risser Grade 3–5, had no clinical neck and back symptoms, and X-rays confirmed no spinal deformities such as scoliosis or kyphosis. The study was approved by the Ethics Committee of Shanghai Tenth People’s Hospital, also informed consent was obtained from each subject or their legal guardians.

### Radiographic measurements

Standard standing whole-spine anteroposterior and later radiographs were obtained for each subject. All the subjects were asked to stand in a natural position, look straightforward, and keep their hands at the level of their clavicles according to the standard position recommended for adults [15, 16]. Regional and global sagittal alignment and spinopelvic alignment parameters were measured, including C7 slope, C2-7 Cobb, C2-S1 sagittal vertical axis (C2-S1 SVA), C7-S1 sagittal vertical axis (C7-S1 SVA), C2-7 plumb line (C2-7PL), T5-12 Cobb, T10-L2 Cobb, L1-S1 Cobb, pelvic incidence (PI), pelvic tilt (PT) and sacral slope (SS). All the radiographic parameters were measured by two experienced spinal surgeons independently.

**Fig. 1:**
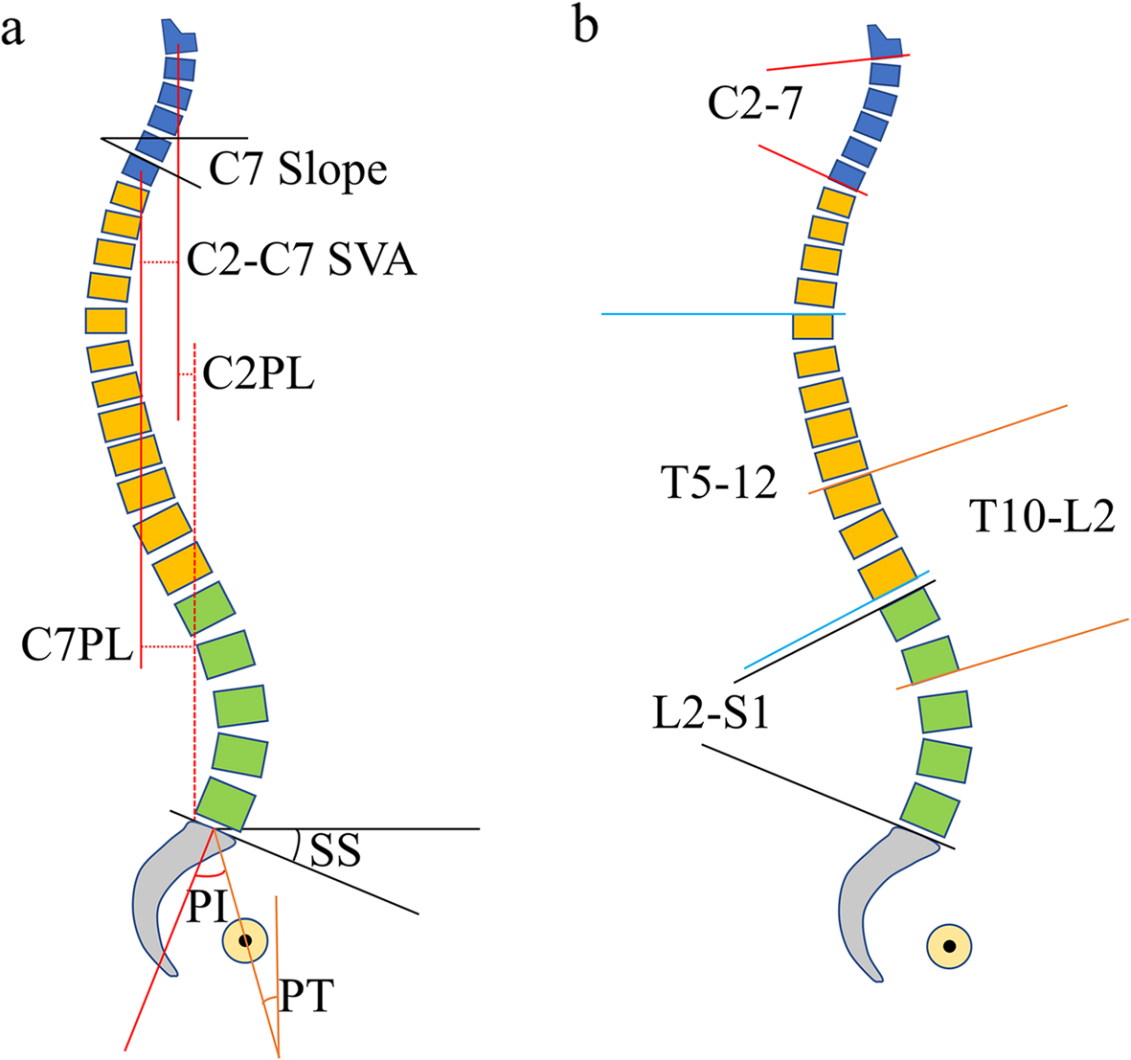
**a** C7 Slope, C2-7 plumb line (PL), C2-S1 Sagittal Vertical Axis (SVA), C7-S1 SVA, pelvic incidence (PI), pelvic tilt (PT), sacral slope (SS). **b** C2-C7 Cobb, T5-12 Cobb, T10-L2 Cobb, L1-S1 Cobb. **a** C7 slope was formed by the horizontal plane and the upper end plate of C7. C2 plumb line and C7 plumb line were defined as the vertical line (PL) drawn from the middle of the body of C2 or C7 vertebral body. C2-S1 SVA and C7-S1 SVA were defined as the horizontal distances from the C2 PL or C7 PL to the posterior superior corner of the sacrum (S1). The deviation of C2 PL and C7 PL was defined as C2-7PL. The PI corresponded to the angle between the perpendicular to the upper S1 level passing through its center and the line connecting this point to the axis of the femoral heads. The PT was defined by the angle between the vertical and the line connecting the center of the sacral endplate to the axis of the femoral heads. The SS was defined by the angle between a line tangent to the upper S1 endplate and horizontal line. **b** C2-7 Cobb angle was measured from the inferior endplate of C2 to the inferior endplate of C7. T5-T12 Cobb angle was measured from the superior endplate of T5 to the inferior endplate of T12 (Fig. 1b). T10-L2 Cobb angle was measured from the superior endplate of T10 to the inferior endplate of L2. L1-S1 Cobb angle was measured from the superior endplate of L1 to the superior endplate of S1

C7 slope was formed by the horizontal plane and the upper endplate of C7 (Fig. 1a). C2-7 Cobb angle was measured from the inferior endplate of C2 to the inferior endplate of C7 (Fig. 1b). C2 plumb line and C7 plumb line were defined as the vertical line (plumb line) drawn from the middle of the C2 or C7 vertebral body (Fig. 1a). C2-S1 SVA and C7-S1 SVA were defined as the horizontal distances from the C2 or C7 plumb line to the posterior superior corner of the sacrum (S1) (Fig. 1a). The deviation of the C2 plumb line and C7 plumb line was defined as C2-7PL (Fig. 1a). T5-12 Cobb angle was measured from the superior endplate of T5 to the inferior endplate of T12 (Fig. 1b). T10-L2 Cobb angle was measured from the superior endplate of T10 to the inferior endplate of L2 (Fig. 1b). L1-S1 Cobb angle was measured from the superior endplate of L1 to the superior endplate of S1 (Fig. 1b). Pelvic parameters including PI, PT, and SS were measured according to the method described in the published paper (Fig. 1a) [6].

**Fig. 2:**
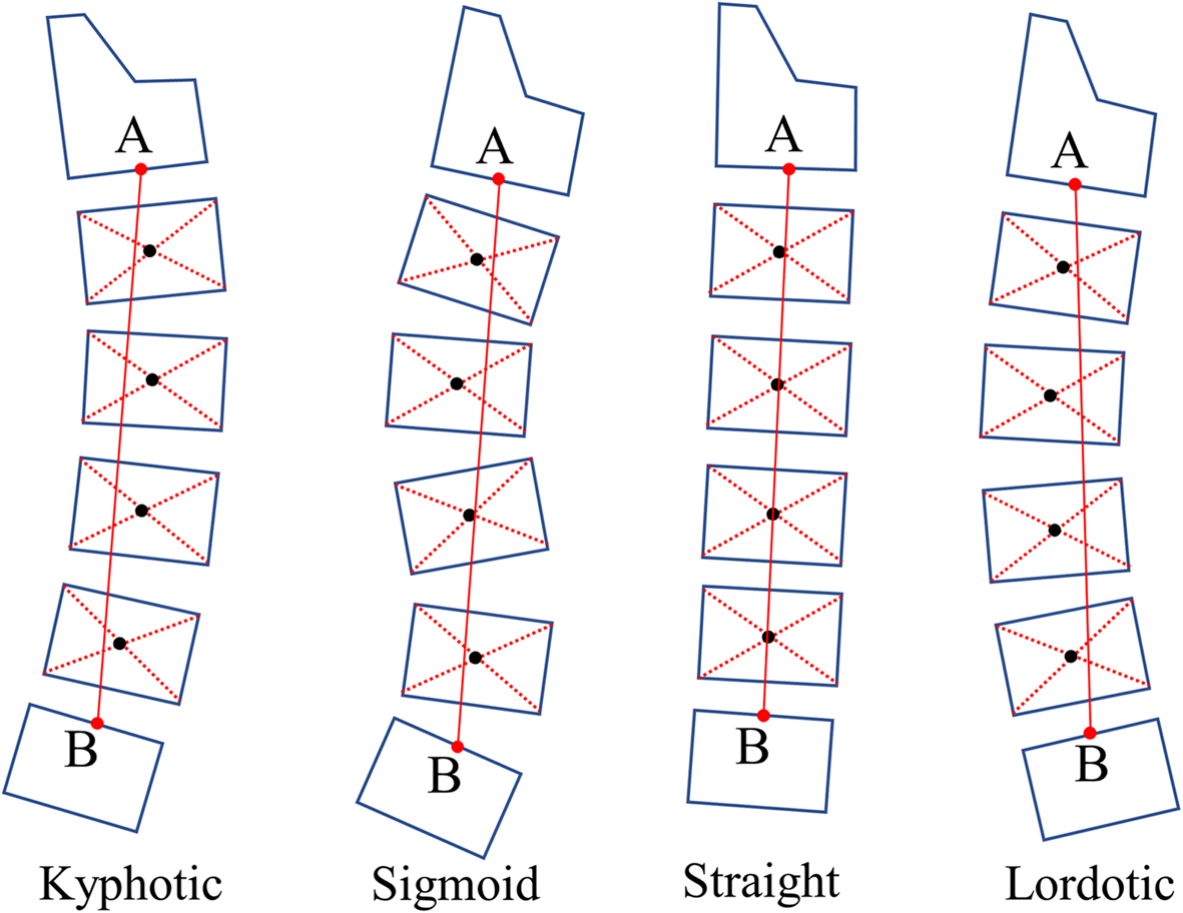
Four types of cervical morphology classified according to a modified method of Toyama et al. A line AB was drawn from the midpoints of the inferior margin of C2 to the midpoints the superior margin of C7. And, the morphology was classified to four types according to the relative positions of the centroids of C3-6 to line AB. Lordotic: all centroids are anterior to AB and the distance between at least one centroid and AB is 2 mm or more; Straight: the distance between line AB and each centroid is less than 2 mm; Sigmoid: some centroids are anterior to and some posterior to line AB and the distance between line AB and at least one centroid is 2 mm or more; Kyphotic: all the centroids are posterior to line AB and the distance between at least one centroid and the AB is 2 mm or more

Besides, cervical morphology was classified by two different methods. One is a modified method by Toyama et al. [17, 18]. In brief, a line AB was drawn from the midpoints of the inferior margin of C2 to the midpoints of the superior margin of C7. And, the morphology was classified into four types according to the relative positions of the centroids of C3-6 to line AB (Fig. 2). Another method is based on the C2-7 Cobb angle < 0° or ≥ 0°, the cervical morphology was classified as lordotic and non-lordotic.

### Statistical analysis

The software of SPSS 22 (SPSS, USA) was used for statistical analysis. Intraclass correlation coefficient (ICC) was used to access the intra-rater and interrater reliability. All the data were shown in the form of mean ± standard deviation (SD) and significance was defined as *P* < 0.05. The correlations of sagittal alignment parameters were examined using the Pearson correlation coefficients, and a two-tailed test was used to test the significance. One-way Anova analysis and independent-sample T-test were conducted to compare the difference between groups.

## Results

All the sagittal alignment parameters are shown in Table 1. The mean value of the C7 Slope was 20.45 ± 8.88°(ranging from-17.50°to 37.50°), and C2-7 Cobb ranged from -51.40° to 24.10° with a mean of -7.72 ± 12.10°, and the mean value of C2-7PL was 13.53 ± 11.63 mm (ranging from -15.44to 37.14 mm). C2-7 Cobb and C7 Slope showed significant differences between the male and female groups. Intraclass correlation coefficient (Table 2) showed good to excellent intra-rater (ICCs ranged from 0.90 to 0.99) and inter-rater (ICCs ranged from 0.85 to 0.95) reliability in measuring the sagittal alignment parameters.

**Table 1:** Details of Sagittal alignment parameters

	Male		Female		*P* value	Total	
	Mean	SD	Mean	SD		Mean	SD
C7 Slope (°)*	23.31	6.72	18.58	9.65	0.005	20.45	8.88
C2-C7 Cobb (°)*	-12.24	11.02	-4.75	11.94	0.001	-7.72	12.10
C2-S1 SVA (mm)	21.88	33.54	11.03	30.87	0.083	15.33	32.25
C7-S1 SVA (mm)*	10.25	28.73	-3.75	28.49	0.013	1.80	29.27
C2-7PL (mm)	11.63	11.99	14.78	11.30	0.164	13.53	11.63
T5-T12 Cobb (°)	24.79	10.37	22.57	8.93	0.231	23.45	9.54
T10-L2 Cobb (°)	3.60	6.87	2.69	7.44	0.518	3.05	7.20
L1-S1 Cobb (°)	51.07	11.03	48.56	11.71	0.262	49.56	11.46
PI (°)*	49.03	11.51	44.52	10.23	0.033	46.30	10.93
PT (°)	11.15	6.67	10.27	34.52	0.524	10.62	7.09
SS (°)	37.88	8.49	34.52	8.92	0.051	35.85	8.87

*Cobb* Angle measured using Cobb method, *SVA* Sagittal Vertical Axis, *PL* Plumb Line, *SD* Standard deviation

“*”: *P* < 0.05

**Table 2:** Reliability analysis of sagittal alignment parameters

	Intra-rater reliability			Inter-rater reliability		
	ICC	95% CI		ICC	95% CI	
C7 Slope	0.99	0.99	0.99	0.95	0.93	0.96
C2-C7 Cobb	0.98	0.97	0.99	0.92	0.90	0.94
C2-S1 SVA	0.90	0.88	0.93	0.86	0.73	0.92
C7-S1 SVA	0.92	0.90	0.94	0.85	0.71	0.91
C2-7PL	0.96	0.95	0.97	0.89	0.86	0.93
T5-T12 Cobb	0.95	0.92	0.96	0.90	0.87	0.92
T10-L2 Cobb	0.93	0.89	0.98	0.89	0.83	0.91
L1-S1 Cobb	0.92	0.87	0.94	0.90	0.88	0.93
PI	0.95	0.93	0.96	0.87	0.79	0.90
PT	0.96	0.95	0.97	0.90	0.87	0.92
SS	0.93	0.91	0.94	0.89	0.86	0.91

*Cobb* Angle measured using Cobb method, *SVA* Sagittal Vertical Axis, *PL* Plumb Line, *ICC* Intraclass correlation analysis, *CI* Confidence interval

### Correlation efficiency analysis

Table 3 showed the correlation efficiency of sagittal alignment parameters. C7 Slope exhibited significant negative correlation with C2-7 Cobb (*r* = -0.544, *P* < 0.001), while significant positive correlation with C2-S1 SVA (*r* = 0.335, *P* < 0.001), C7-S1 SVA (*r* = 0.310, *P* = 0.001), T5-12 Cobb (*r* = 0.236, *P* = 0.013), respectively. C2-7 Cobb showed significant negative correlation with C7-S1 SVA (*r* = -0.255, *P* = 0.007) but positively related with C2-7PL (*r* = 0.319, *P* = 0.001). C2-7PL showed significant positive correlation with C2-S1 SVA (*r* = 0.405, *P* < 0.001). Cervical reginal sagittal alignment parameters including C7 Slope, C2-7 Cobb, C2-7 PL showed no significant correlation with lumbosacral parameters (L5-S1 Cobb, PI, PT, SS).

**Table 3:** Correlation efficiency of sagittal alignment parameters

Parameters	C7 Slope	C2-7 Cobb	C2-S1 SVA	C7-S1 SVA	C2-7 PL	T5-12 Cobb	T10-L2 Cobb	L1-S1 Cobb	PI	PT	SS
C7 Slope	1.000	-0.544**	0.335**	0.310*	0.148	0.236*	0.027	0.056	0.181	0.154	0.110
		0.000	0.000	0.001	0.122	0.013	0.776	0.562	0.057	0.106	0.251
C2-7 Cobb		1.000	-0.092	-0.251*	.379**	-0.210*	-0.096	-0.023	-0.021	0.012	-0.043
			0.339	0.008	0.000	0.027	0.318	0.809	0.829	0.904	0.651
C2-S1 SVA			1.000	0.933**	0.424**	-0.090	0.019	-0.104	0.266*	0.170	0.192*
				0.000	0.000	0.346	0.844	0.276	0.005	0.075	0.044
C7-S1 SVA				1.000	0.070	-0.132	0.010	-0.144	0.253*	0.147	0.196*
					0.465	0.167	0.915	0.132	0.007	0.124	0.040
C2-7 PL					1.000	0.082	0.027	0.073	0.102	0.101	0.040
						0.392	0.781	0.449	0.289	0.292	0.677
T5-12 Cobb						1.000	0.156	0.486**	0.086	-0.050	0.177
							0.102	0.000	0.368	0.604	0.062
T10-L2 Cobb							1.000	-0.255*	-.282*	-0.074	-.327**
								0.007	0.003	0.442	0.000
L1-S1 Cobb								1.000	0.585**	-0.066	0.828**
									0.000	0.493	0.000
PI									1.000	0.599**	0.728**
										0.000	0.000
PT										1.000	-0.073
											0.444
SS											1.000

“*”: *P* < 0.05

“**”: *P* < 0.001

### Cervical morphology and sagittal alignment parameters

All the subjects were divided into three groups according to cervical morphology, Lordotic group, Straight or Sigmoid group, and Kyphotic group, and the data were shown in Table 4. According to the modified method, there were 37 lordotic (33.3%), 39 straight (35.1%), 16 sigmoid (14.4%) and 19 kyphotic cases (17.1%). The mean value of the C7 slope in the Lordotic group is 26.91 ± 5.40°, which is significantly different from the Straight or Sigmoid group (17.91 ± 8.84°, *P* < 0.05). C2-7 Cobb showed significant differences between the three groups (*P* < 0.05) and C2-C7PL showed a significant difference between Lordosis and Kyphotic group (*P* < 0.05).

**Table 4:** Comparison of sagittal alignment parameters among three groups classified by modified method

	Lordotic		Straight/Sigmoid		Kyphotic		*P* value	Sig		
N	37		55		19					
	Mean	SD	Mean	SD	Mean	SD				
C7 Slope (°)	26.91	5.4	17.91	8.84	15.24	7.43	0.000	*	**	
C2-C7 Cobb (°)	-18.26	10.94	-4.81	7.64	4.39	8.47	0.000	*	**	***
C2-S1 SVA (mm)	19.63	35.43	12.51	28.19	15.12	37.37	0.587			
C7-S1 SVA (mm)	9.72	31.3	-1.49	23.72	-4.1	37.29	0.124			
C2-7PL (mm)	9.91	13.11	14	11.01	19.23	7.53	0.015		**	
T5-T12 Cobb (°)	25.75	9.11	22.22	9.92	22.52	8.89	0.198			
T10-L2 Cobb (°)	4.19	7.36	2.63	6.43	2.08	8.98	0.488			
L1-S1 Cobb (°)	50.61	12.12	49.34	10.68	48.13	12.75	0.733			
PI (°)	48.88	10.98	45.24	10.97	44.38	10.36	0.207			
PT (°)	12.62	6.53	8.71	7.55	12.24	5.36	0.018	*		
SS (°)	36.82	8.58	36.28	7.99	32.72	11.37	0.232			

“*”: Lordosis group vs Straight/Sigmoid group, *P* < 0.05

“**”: Lordosis group vs Kyphotic group, *P* < 0.05

“***”: Straight/Sigmoid group vs Kyphotic group, *P* < 0.05

### C2-7 Cobb and sagittal alignment parameters

All the subjects were divided into two groups according to C2-7 Cobb, the Lordotic group (C2-7 Cobb < 0, *n* = 80), and the Non-Lordotic group (C2-7 Cobb ≥ 0, *n* = 31), and the data was shown in Table 5. Intergroup analysis showed that the C7 Slope (Lordosis vs Non-lordotic: 22.52 ± 8.94°vs 15.14 ± 6.18°, *P* < 0.001), C2-7PL (Lordosis vs Non-lordotic: 11.86 ± 12.49 mm vs 18.05 ± 7.3 mm, *P* = 0.001) and C7-S1 SVA were significantly different between the two groups.

**Table 5:** Comparison of sagittal alignment parameters between lordotic and non-lordotic group

	Lordotic (C2-7 Cobb < 0)		Non-Lordotic (C2-7 Cobb ≥ 0)		*P* value
N	80		31		
	Mean	SD	Mean	SD	
C7 Slope (°)*	22.52	8.94	15.14	6.18	0.000
C2-C7 Cobb (°)*	-13.05	9.60	6.03	4.85	0.000
C2-S1 SVA (mm)	16.96	31.92	11.12	33.23	0.395
C7-S1 SVA (mm)*	5.23	27.75	-7.07	31.65	0.047
C2-7PL (mm)*	11.73	12.52	18.19	7.21	0.001
T5-T12 Cobb (°)	24.36	9.67	21.11	8.95	0.108
T10-L2 Cobb (°)	3.44	7.16	2.06	7.34	0.375
L1-S1 Cobb (°)	49.63	12.10	49.37	9.80	0.915
PI (°)	46.08	11.52	46.89	9.41	0.726
PT (°)	10.55	7.42	10.79	6.25	0.874
SS (°)	35.78	8.82	36.03	9.12	0.894

“*”: *P* < 0.05

## Discussion

### Lordotic or non-lordotic?

Although it is well accepted that lordosis is the natural cervical alignment of the cervical spine, the definition of “normal” cervical spine morphology remains controversial. Yu et al. [7] included 120 cases of asymptomatic subjects (mean age 23.2 ± 6.3 years) showed that only 28.3% (34/120) of the subjects with lordotic cervical alignment. Similarly, Kim et al. [2] showed around one-fourth (26.3%) of asymptomatic adult volunteers have kyphotic cervical alignment. These data are from asymptomatic adult volunteers, more interestingly, K. Abelin-Genevois et al. reported data from a normal pediatric Caucasian population, which also found a high prevalence of kyphotic or straight morphology [15]. In this study, we found there were only 33.3% of asymptomatic adolescent subjects with cervical lordosis according to the modified method, and around 72.1% of subjects with cervical lordosis according to C2-7 Cobb. More importantly, all the subjects we included were adolescence ranging from 12 to 20 years old with Risser sign ≥ 3, it allows us to better understand the morphological characteristics of the cervical spine after it has reached a stable state of natural development. It provides more reliable evidence that the cervical spine morphology of asymptomatic adolescents varies widely, from lordotic to kyphotic. Therefore, it is more reasonable to diagnose a cervical spine alignment that fails to achieve the horizontal gaze or causes symptoms like neck pain as pathological, rather than a kyphotic cervical alignment itself.

Notably, these findings do support there is a quiet percentage of kyphotic cervical alignment in asymptomatic populations, but do not suggest cervical surgery planning should allow the cervical alignment to be kyphotic after surgery. Villavicencio et al. [19] showed that those patients who are maintained or more lordotic of the fused segment alignment postoperatively had better surgical outcomes than those who became more kyphotic. Brooke et al. [20] showed increased NDI scores were correlated with cervical kyphosis.

### Which cervical regional sagittal alignment parameters should be considered pre-operatively?

It is gradually becoming a consensus that not only regional but global spinal alignment should be taken into account for the surgical treatment of the cervical spine. In clinical practice, however, sometimes only lateral radiographs of the cervical spine are obtained pre-operatively, rather than the global spine, especially in some developing countries. So, what cervical regional sagittal alignment parameters can reflect the global sagittal balance? In this study, we showed that the C7 slope showed a significant correlation with global spine sagittal alignment parameters C2-S1 SVA and C7-S1 SVA. And we provided evidence that C2-7PL is the key to restoring cervical lordosis.

#### C7 slope

**Fig. 3:**
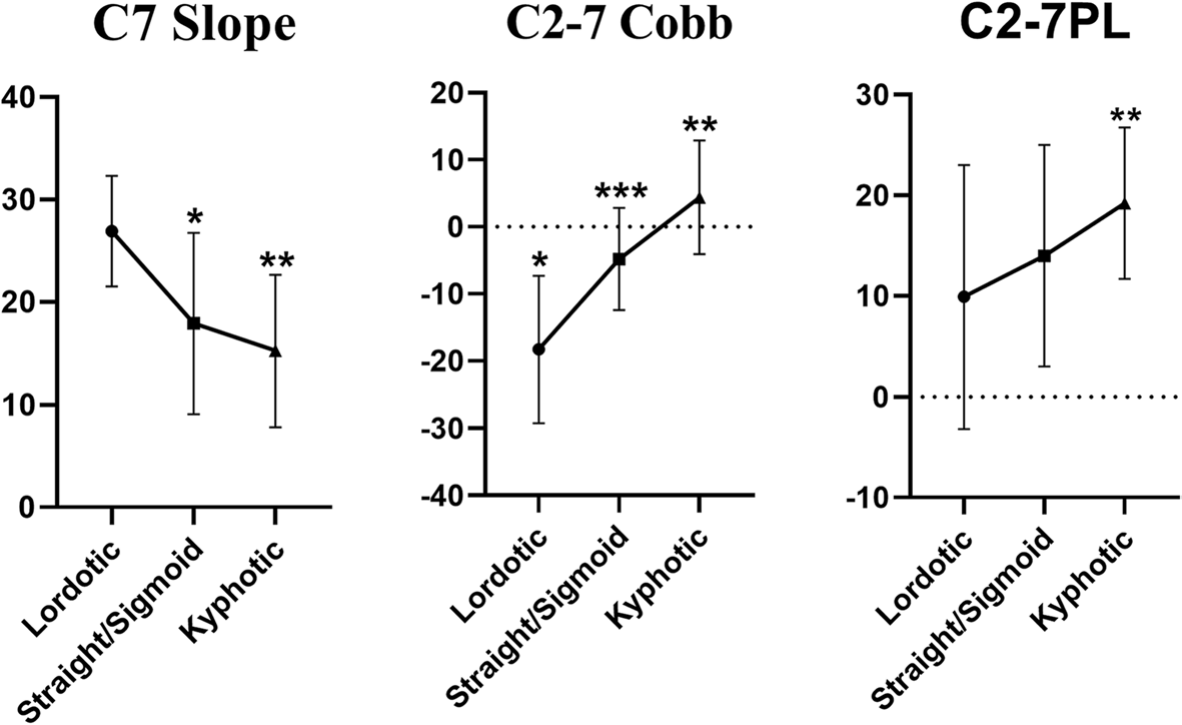
Comparison of C7 slope, C2-7 Cobb, C2-7PL among three groups classified by modified method. “*”: Lordosis group vs Straight/Sigmoid group, *P* < 0.05; “**”: Lordosis group vs Kyphotic group, *P* < 0.05; “***”: Straight/Sigmoid group vs Kyphotic group, *P* < 0.05

C7 slope was formed by the horizontal plane and the upper endplate of C7, and it is the bridge between the cervical and thoracic spine. Tamai et al. showed that C7 slope was significantly correlated with cervical tilt, cranial tilt, neck tilt, C2-7 Cobb, and highly correlated with T1 slope, which is another important key factor of cervical sagittal balance [21]. In this study, the C7 slope was significantly different between groups. With the cervical morphology changed from lordosis to kyphosis, the C7 slope decreased while cervical lordosis and C2-7PL increased (Fig. 3). Correlation analysis showed that the C7 slope was significantly correlated with C2-S1 SVA and C7-S1 SVA, which are both important parameters predicting global sagittal alignment balance [3, 10], indicating that the C7 slope can be used to predict the global sagittal balance when lacking whole spinal lateral radiographs.

#### C2-7PL

There are two similar definitions of C2-7PL, one is the horizontal distances from the C2 plumb line to the posterior superior corner of C7, and another relatively simple way is the deviation of the C2 plumb line and C7 plumb line. In this study, we use the latter method and C2-7PL was ranging from -15.44 to 37.14 mm. Correlation analysis showed that C2-7PL was significantly positively correlated with C2-7 Cobb. With increasing C2-7 Cobb, the lordosis gradually disappeared and C2-7PL increased. A previous study showed that the forehead posture increased O-C2 lordosis and decreased C2-C7 lordosis using CT scan reconstruction [16]. Penning also reported that during forwarding translation of the head without flexion, the upper cervical spine went into extension and the lower cervical spine went into flexion [22]. All these findings indicated that changing C2-7PL can help to restore cervical lordosis.

Relatively, a slight extension of the head didn’t change a reversed cervical curve into a cervical lordosis as measured on lateral cervical radiographs [23], indicating that simply maintaining the head in a tilt position during traction therapy may not help restore the cervical lordosis and that consideration should be given to how to restore C2-7PL.

### What else we can learn from the radiographic analysis of 111 asymptomatic adolescents?

It is worth thinking about what method to use to define and classify the morphology of the cervical spine. C2-7 Cobb has been considered as a key parameter in cervical sagittal alignment, being used as a measurement of lordosis because of its good intra- and interrater reliability and feasibility [11, 24]. We divided all the subjects into the lordotic group (C2-7 Cobb < 0°) and the non-lordotic group (C2-7 Cobb ≥ 0°). There were 73% of subjects with lordotic cervical sagittal alignment, this percentage was similar to what Ella Been et al. [14] showed in a previous study, that there were 71% of the children with lordotic cervical spine in the children’s group (aging from 6–19 years old). However, the percentage is much higher than that classified by the modified method of Toyama et al. [17, 18], in which only 33.3% of subjects in the Lordosis group. Most recently, Sohrab Virk et al. [25] developed a new method to better define the morphology of the cervical spine. All these indicate that combining different methods to evaluate the cervical sagittal alignment can get a more comprehensive understanding of cervical sagittal morphology.

There are differences in the cervical spine morphology between gender. Previously, Yasutsugu Yukawa et al. [26] reported that C3-7 cervical lordosis showed a significant difference in 20–29 years old asymptomatic individuals. Kuang-Ting Yeh et al. [27] found that C7 Slope and C2-7 SVA were significantly different in asymptomatic adults. In this study, we showed that C2-7 Cobb, and C7 Slope showed significant differences between the male and female groups. All these findings gave evidence that there are differences in the cervical sagittal alignment between males and females, indicating sex differences should be considered pre-operatively.

In addition, we found cervical regional sagittal alignment parameters including C7 Slope, C2-7 Cobb, and C2-7 PL showed no significant correlation with lumbosacral parameters (L5-S1 Cobb, PI, PT, SS). Le Huec et al. [21] also showed a poor correlation between the pelvic parameters and the cervical parameters.

There are several limitations of this study. The total number of subjects we included is relatively small. Another limitation is that we didn’t get the data like HRQOL and NDI from the subjects, which could better quantify the relation between sagittal alignment parameters and life quality. On the other hand, although all the subjects we included were currently asymptomatic, there were no following-up data to investigate the relationship between cervical morphology and degeneration. Also, there are some studies included some angles between the head and cervical spine like Occipito C2 angle, spino-cranial angle [15, 28]. Since the subjects in this study are all asymptomatic and can gain a horizontal gaze, so we didn’t include these parameters. However, given the fact that our data were from asymptomatic adolescent subjects, making it is worthy to better understand the morphology of cervical sagittal alignment.

In summary, our study demonstrated that there are different types of cervical morphology in asymptomatic adolescents, with a wide range of C2-7 Cobb angles, from lordosis to kyphosis. C7 slope is an important predictor of global sagittal balance and C2-7PL is a key parameter for restoring cervical lordosis, which should be considered pre-operatively and for conservative treatment. The classified method will make a different conclusion about cervical morphology, a more comprehensive understanding of cervical morphology can be gained by combining the different methods. The cervical regional sagittal alignment parameters are not correlated with lumbosacral parameters, and C2-7 Cobb, C7 Slope showed significant differences between males and females.

## Data Availability

Data available on request due to privacy/ethical restrictions. To access it, please email Dr. Ni: nihaijianch@163.com.
